# Evaluating Anthropometric Indices for Malnutrition Assessment in Older Adults: Scoping Review

**DOI:** 10.1007/s13668-025-00654-z

**Published:** 2025-05-07

**Authors:** Serife Akpinar Senture, Eda Koksal

**Affiliations:** Faculty of Health Science, Department of Nutrition and Dietetic, Gazi University, Emek, Bişkek Main St. 6. St No: 2, Çankaya, Ankara 06490 Turkey

**Keywords:** Malnutrition, Older adults, Anthropometric indices, Undernutrition, Obesity, BRI (Body Roundness Index), BAI (Body Adiposity Index), ABSI (A Body Shape Index), Conicity Index (CI), Waist-adjusted Weight Index (WWI), Abdominal Volume Index (AVI), Demiquet, Mindeks

## Abstract

**Purpose of Review:**

The demographic transition in both global and national populations indicates a growing trend in the older population. This burgeoning older population brings about constraints marked by the effective presence of malnutrition alongside functional and physiological changes. Conducting screenings and assessments to prevent malnutrition and enable early intervention is crucial. In epidemiological and clinical studies, these screenings and assessments are often conducted through anthropometric measurements and a range of indices derived from them, encompassing both traditional ones like BMI (Body Mass Index) and newer ones like BRI (Body Roundness Index), BAI (Body Adiposity Index), ABSI (A Body Shape Index), CI (Conicity Index), WWI (Waist adjusted Weight Index), AVI (Abdominal Volume Index), Demiquet, and Mindeks. There is a lack of scrutiny regarding studies comparing these indices based on the type of malnutrition in the older population, hence this review aims to address the gap in the literature.

**Recent Findings:**

Analysis of existing research reveals that BRI and waist-to-height ratio serve as predictors of obesity-related body fat quantity/distribution and metabolic risks, while WWI stands out in sarcopenic obesity, and Demiquet and Mindeks are prominent indices for screening inadequate nutrition.

**Summary:**

Choosing anthropometric indices that do not include height in the older populations can have certain advantages. However, the selection of these indices should depend on the specific variable being assessed or screened such as obesity, malnutrition, or sarcopenic obesity.

## Introductıon

With increased longevity and a decline in fertility rates, the rate of population aging is accelerating, imparting an irreversible trend toward global aging [1]. Indeed, World Health Organization (WHO) data from 2020 reports that the number of individuals aged 60 and over has reached 1.4 billion, surpassing the number of children under the age of five. Moreover, projections indicate that by 2050, the prevalence of the older population is expected to rise to 22% [2]. This increase in the older population, coupled with age-related degenerative changes, leads to a range of limitations that negatively impact the quality of life as a result of acute and/or chronic diseases [3]. One of the most common problems encountered in old age is malnutrition, which is a significant factor that can affect the etiology and prognosis of diseases and consequently increase healthcare costs [4]. In this context, it is crucial to conduct screenings and assessments related to malnutrition in the older adults and plan interventions when necessary.

Malnutrition is defined as a condition resulting from inadequate intake or utilization of nutrients, leading to changes in body composition and subsequently reducing physical and mental functions, which exacerbates clinical outcomes in the presence of disease [5]. The prevalence of malnutrition among older adults is estimated to be close to 50%. Both undernutrition and overnutrition (obesity) fall within the scope of malnutrition [6]. Indeed, just as undernutrition impacts life expectancy, so does overnutrition. It is reported that being overweight reduces life expectancy by three years, while obesity is associated with a 6–7 year reduction in life expectancy. Additionally, obesity increases the risk of premature death by 81% in men and 115% in women [7].

Obesity is a term that refers not merely to excess body weight but to an excess in body fat percentage and distribution [8]. Changes in body composition in the older adults are characterized by a tendency towards increased body fat [9, 10]. While studies on the older adults suggest that having upper-limit body fat and a slight increase may be protective against diseases, excessive body fat is undeniably associated with increased morbidity and mortality risks [11]. Recent meta-analyses have shown that higher fat mass is effective in coping with diseases only when muscle mass is also high [12]. The increase in adipose tissue and the decrease in muscle tissue is termed sarcopenic obesity, and age-related changes in body composition (decrease in muscle mass, increase in fat mass, decrease in muscle strength, and physical performance) can occur [13]. Early diagnosis and treatment are mandatory; however, there is no standard diagnostic criterion for sarcopenic obesity, which is primarily defined by a decrease in muscle mass and strength and an increase in fat mass [14]. High serum leptin levels observed in obesity may also be a cause of low muscle strength and quality. Sarcopenic obesity is common among the older adults, affecting one in ten older adults [12, 15].

The prevalence of undernutrition among older adults is generally reported to be below 10% among those living independently, while it rises to up to two-thirds in hospitalized older patients [16]. However, these prevalence estimates vary significantly depending on the population considered, the healthcare setting, and the tool used for assessment [17]. The Global Leadership Initiative on Malnutrition (GLIM) criteria have been established for the universally accepted diagnosis of malnutrition. These two-stage criteria, which include phenotypic and etiological criteria, aim to standardize the identification of malnutrition and underscore the importance of detecting malnutrition and implementing necessary interventions [18]. Accurate assessment of height and body weight is essential for identifying the presence of phenotypic criteria.

In evaluating malnutrition, various tools for assessing nutritional status, including anthropometric and laboratory methods, biochemical markers, clinical symptoms, dietary intake, and environmental/ecological factors, can be considered [19]. Among these methods, anthropometric measurements and the indices derived from them are used as non-invasive, practical, easy, and inexpensive tools for screening and assessing nutritional status in both field and clinical studies [6]. Anthropometric indices not only help in determining the risk of malnutrition but also in grading cardiometabolic risks, predicting alarm and action situations, and enabling the monitoring of individuals after nutritional intervention [18, 20–22]. While the Body Mass Index (BMI) is the most widely used anthropometric index for screening and monitoring nutritional status, other indices such as Demiquet, Mindex, Waist-to-Height Ratio (WHtR), Body Roundness Index (BRI), Body Adiposity Index (BAI), Abdominal Volume Index (AVI), A Body Shape Index (ABSI), Conicity Index (CI), and Weight-Adjusted Waist Index (WWI) are also associated with nutritional status and health [6, 23].

This review focuses on the anthropometric indices used for screening malnutrition in older individuals and their associations with health. Relevant studies from the past five years were reviewed, using databases such as Google Scholar, PubMed, and Science Direct, with search terms including"anthropometric indices used in older adults,""Body Roundness Index (BRI) and older adults,""Body Adiposity Index (BAI) and older adults,""Abdominal Volume Index (AVI) and older adults,""Conicity Index (CI) and older adults,""A Body Shape Index (ABSI) and older adults,"and"WWI and older adults."Studies comparing these indices were examined, and evaluations were made based on conditions included in the definition of malnutrition.

## Anthropometric Indices Used in the Older Adults

### Traditional Anthropometric Indices (BMI and WHtR)

Body Mass Index (BMI), obtained by dividing an individual’s body weight in kilograms by the square of their height in meters (kg/m^2^), is one of the indicators of nutritional status [24]. It is a practical screening tool for identifying obesity, but it should not be considered a definitive tool due to its inadequacy in assessing body fat distribution. Both the WHO and American health authorities have reported that the same cut-off points for defining obesity are valid for all age groups [25].

Due to changes in body composition observed with advancing age, malnutrition has been found to be associated with the ability to cope with chronic diseases and mortality risk [5]. A meta-analysis study examining 32 studies from 1990 to 2013 used a reference BMI range of 23.0–23.9 and found that older adults with a BMI of 21.0–21.9 had a 12% higher risk of mortality, and those with a BMI of 20.0–20.9 had a 19% higher risk of mortality. Additionally, older adults with a BMI above 33 kg/m^2^ had an 8% higher mortality risk [26].

Although BMI is widely used for screening nutritional status, it does not provide information about body fat distribution and overlooks body composition changes observed with aging, even when age-specific cut-off points are used [27]. BMI has been shown to overestimate the number of underweight, normal, and overweight individuals and underestimate the number of obese individuals among the older adults. In a study involving various ethnicities (4984 individuals aged 60 and over), the accuracy of BMI in classifying obesity was compared with dual-energy X-ray absorptiometry (DXA). When a BMI of 30 kg/m^2^ and above was considered obese, 41% of men and 45% of women were correctly classified. When the cut-off was lowered to 25 kg/m^2^ and above, 80% of men and 78% of women were correctly classified. The study concluded that BMI is not an optimal indicator of adiposity in the older adults [28].

Waist circumference measurements are used to assess the risk of metabolic diseases associated with obesity and are thus important for evaluation in the elderly (older adults) [29]. The WHtR, obtained by dividing waist circumference (cm) by height (cm), has been found to be the best indicator of biochemical parameters of cardiovascular diseases, such as dyslipidemia and hyperglycemia, in middle-aged individuals (50 years and older). Similarly, it has been identified as a good predictor of metabolic syndrome in the older adults [30]. The WHtRwas found to have the highest predictive power for metabolic syndrome in older people (AUC = 0.786; 95% CI: 0.76–0.81) [31].

### New Anthropometric Indices (BRI, BAI, ABSI, CI, WWI, AVI, Demiquet, Mindex)

Several new anthropometric indices have been developed for estimating body composition. Among these, BRI, BAI, ABSI, CI, and AVI were developed for adults but are also used for the elderly. Demiquet and Mindex are indices specifically developed for the elderly.

The Body Roundness Index (BRI), developed to estimate body fat and visceral adipose tissue percentage. It has been reported that this index improves the prediction of fat distribution, i.e., obesity risk [32, 33]. Although calculating and interpreting the WHtR and BMI are simple, BRI is suggested to be a better indicator of physical health [34]. Using the NHANES database, regression models for BRI, including anthropometric measurements (waist circumference, hip circumference, height, etc.), total fat (%), and visceral adiposity (%), were found to perform better than other indices in predicting body fat percentage and visceral adipose tissue percentage (R^2^ for body fat percentage = 0.88, R^2^ for visceral adipose tissue percentage = 0.69) [32].

$$BRI=364.2-365.5[1-{\pi }^{2}\; {WC}^{2}\; ({m}^{2})\; {height}^{2}\; ({m}^{2}){]}^{1/2}(WC: waist \ circumference)$$

The Body Adiposity Index (BAI), calculated using hip circumference and height measurements, was developed to estimate body adiposity and has shown a high correlation with body fat percentage measured by DXA in the adult age group (r = 0.85) (r = 0,85) [35]. Other studies have found that body fat percentage values obtained by Bioelectric impedence analyses (BIA) and those calculated by BAI were consistent both in adults aged 19–64 (r = 0.479) and in the older adults aged 60 and over (r = 0.79). It is suggested that BAI can be used to estimate body fat percentage in both adults and older people [36, 37].

$$BAI= HC\; (cm)/{Height }^{1.5}(m)\; (HC: Hip \ Circumference)$$

Different cut-off points specific to age and gender are used for evaluating body fat percentage derived from BAI. The cut-off points used are presented in Table 1 [38].

**Table 1:** Evaluation of body fat percentage estimates derived from BAI

	≥ 60 aged	
	Male	Female
Undernutrition	< 13%	< 25%
Normal	13–25%	25–38%
Overweight	26–31%	39–43%
Obesity	> 31%	> 43%

A new approach that standardizes waist circumference measurement based on height and BMI, known as the A Body Shape Index (ABSI), has been proposed for determining abdominal obesity [39]. ABSI has been shown to accurately reflect visceral fat [40, 41] and is a good indicator of skeletal muscle loss [42–44]. Furthermore, high ABSI values are positively correlated with both morbidity and mortality risk [45].

$$ABSI= WC/\; ({BMI}^\frac{2}{3} \times {Height}^\frac{1}{2})\; (WC: waist \ circumference,\; BMI: Body \ mass \ index)$$

The Conicity Index (CI), developed for screening abdominal obesity, is an index that includes measurements of body weight, height, and waist circumference [46]. Conicity Index is most associated with cardiometabolic diseases and parallels body fat percentage [47, 48].

$$CI ={ 0.109}^{-1}WC\; (m)\; (BW\; (kg)/ Height\; (m){)}^{-1/2} (WC: waist \ circumference, BW: Body \ Weight)$$

The Abdominal Volume Index (AVI) is suggested as a new obesity index that can be used to estimate abdominal fat rather than total visceral body fat [49]. It has been reported that body fat percentage, body fat mass, and BMI are highly correlated with AVI [50].

$$AVI = [2{WC}^{2}\; (cm)+ 0.7\; (WC-HC{)}^{2}]/1000\; (WC: waist \ circumference, HC: hip \ circumference)$$

The WWI was developed considering the inadequacy of BMI in differentiating low muscle mass in the obesity paradox and the waist circumference reflecting body fat distribution. High waist circumference and WHtRvalues may be due not only to high fat mass but also to high muscle mass. This index, which represents waist circumference and is negatively correlated with height, essentially involves standardizing waist circumference for body weight [51]. This index, attributed as a new obesity index, is reported to be associated with survival and sarcopenic obesity in populations aged 60 and over [52, 53].

$$WWI = WC\; (cm)/\surd BW (\surd kg)\; (WC: waist \ circumference, BW: Body \ Weight )$$

It is not appropriate to apply BMI cut-off points for malnutrition and obesity used in adulthood directly to the older population [54, 55]. Evaluation of height in older individuals becomes difficult due to reasons such as kyphoscoliosis, disc slippage, and inability to stand. Demi-span measurement is advantageous for older individuals who cannot stand or whose height measurement cannot be taken (osteoporosis, disc slippage, kyphoscoliosis). There is a stable relationship between arm span and body weight in older individuals with normal morphology [56]. Considering these factors, indices alternative to BMI (Demiquet, Mindex) have been developed for evaluating nutritional status in the (older adults)elderly. A study by Lehman et al. found that body weight is directly proportional to arm span in women and the square of arm span in men [57]. Therefore, Demiquet is recommended for the male gender and Mindex for the female gender.

$$Demiquet\; (Male)= BW\; (kg) / {Demispan}^{2}\; \left({m}^{2}\right)\;(BW: Body \ Weight)$$

$$Mindex\; (Female)= BW\; (kg) / Demispan\; \left(m\right)\;(BW: Body \ Weight)$$

The anthropometric measurements included in the anthropometric indices used in the older adults are formulated above and summarized in Table 2. Demiquet, Mindex, AVI, and WWI do not include height measurement, which can be prone to errors in the older adults. WHtR, BRI, ABSI, CI, AVI, and WWI indices are advantageous for estimating body fat distribution as they include waist circumference measurements.

**Table 2:** The anthropometric measurement contents of anthropometric indices

Anthropometric Index Type	Body weight	Height	WC	HC	Demispan
BMI	✓	✓			
WHtR		✓	✓		
BRI		✓	✓		
BAI		✓		✓	
ABSI	✓	✓	✓		
CI	✓	✓	✓		
AVI			✓	✓	
WWI	✓		✓		
Demiquet/Mindex	✓				✓

*WC *Waist circumference, *HC *Hip circumference, *BMI *Body mass index, *WHtR *Waist to Height ratio, *BRI *Body roundness index, *BAI *Body adiposity index, *ABSI *A body shape index, *CI *Conicity İndex, *AVI *Abdominal Volume İndex, *WWI *Weight adjusted waist index

## Studies on Anthropometric Indices for Assessing Malnutrition Risk

Both traditional (BMI, WHtR) and new anthropometric indices (BRI, BAI, ABSI, CI, WWI, AVI, Demiquet, Mindex) can be used to screen for malnutrition and estimate body composition. While there is a variety of studies in the literature regarding these indices, studies that evaluate and compare these indices specifically in the elderly (older) population are limited. Table 3 provides details of studies conducted in the last five years that have evaluated and compared anthropometric indices in the context of obesity, related diseases, and malnutrition.

**Table 3:** Comparative studies on anthropometric indices in the older adults over the last five years

Malnutrition type	Author, Year	Study Design	Country	Examined indices	Results
Obesity and linked disease	Khosravian et al. 2021 [31]	Cross-sectional, n: 1488 (60–92 aged) mean age: 69,7 ± 7,30 year	Iran	BMI WHtR* AVI CI	**WHtR is the most significant parameter for body fat distribution**, followed by BMI and AVI respectively
	Marzban et al. 2022 [58]	Cohort, n:3000, mean age:67,75 ± 7,10 year	Iran	BMI WHtR AVI BRI*	BRI has the highest discriminatory power compared to BMI and WHtR
	Li et al. 2023 [59]	Cross-sectional, n:9457(45–98 aged) 72,26%, 45–64 year; 27,73%, 65 + year	China	BMI WHtR ABSI BRI* VAI	**BRI is a good indicator of metabolic syndrome**, which is directly associated with obesity in both genders
	Rico‐Martín et al. 2020 [60]	Systematic review and meta-analysis, n:14 study	Populations of various nationalities and ethnic groups	BMI WHtR ABSI BRI*	**BRI is a good predictor of metabolic syndrome** and is superior to other indices
	Geng et al. 2022 [61]	Cohort, n: 75,028 45–85 aged	China	BRI* WHtR* BMI*	**Indices that evaluate central obesity in addition to BMI are important for identifying multimorbidity**. The risk of developing multimorbidity increases by 82% with high BMI, 39% with high BRI, and 28% with a high WHtR
	Ramírez-Vélez et al. 2019 [62]	Cross-sectional, n: 1502 (60 + aged) Mean age: 70 ± 7,6 year	Spain	BMI WHtR* ABSI BRI* CI	**BRI and WHtR** (both with an AUC of 0.77 for both sexes and higher cut-off points in women compared to men) **are appropriate screening tools for assessing MetS risk**
	Geraci et al. 2019 [63]	Cross-sectional, n:468 30–80 aged	Italy	ABSI* BRI BMI WHtR	While **BRI and BMI are associated with carotid thickness**, ABSI shows a stronger correlation with carotid atherosclerosis than traditional indices (p = 0.001)
	Ding et al. 2023 [64]	Cohort (7,93 year followed), n: 71,166 > 45 aged Mean age: 63,06 year (56,48–77,78)	China	BRI* BMI	**There is a U-shaped relationship between BRI and cardiovascular disease** (CVD)-related mortality, with higher BRI being positively associated with deaths from CVD
	Xu et al. 2021 [65]	Cross-sectional, n: 17,360 18–95 aged, (grouped for those over 60 year of age)	China	BMI WHtR BRI* ABSI BAI	In the 60 years and older age group, **BRI demonstrates high sensitivity and specificity in predicting cardiometabolic risk factors** (72% and 63%, respectively)
	Cancela-Carral et al. 2023[66]	Retrospective, n:608 65 + aged Mean age: 68,05 ± 5,43 year	European population (Portugal,Bulgaria, Italy,Slovenia,Hungary, Spain)	BMI* WHtR BAI CI	**BMI has the highest correlation with body fat percentage and cardiometabolic risk** (r = 0.612), followed by BAI (r = 0.556) and WHtR (r = 0.521)
	Gui et al. 2024 [67]	Cross-sectional, n: 9488 > 45 aged (65 + n:1356)	China	WHtR* ABSI BRI* CI	**WHtR and BRI is an effective method for screening high-risk groups for obesity-related hypertension** in middle-aged and older people, Waist/Height is more practical
	Tang et al. 2021 [68]	Cross-sectional, n: 3077 65 + aged Mean age: 70.92 ± 5.84 year	China	ABSI BRI* BMI WHtR *	Geriatric individuals with higher ABSI, BRI and WHtR values are at higher risk for organ injuries due to obesity-related hypertension, **in women, BRI and WHtR are significantly more associated than other anthropometric indices**
	Dong et al. 2023 [69]	Cross-sectional, n: 944,760 65 + aged	China	BMI WHtR* BRI* ABSI CI	All anthropometric indices **except ABSI** show a significant, non-linear positive association with hypertension risk by gender (pnonlinear < 0.001) But the **BRI and WHtR relationship is stronger**
	Zhang et al. 2021 [70]	Cross-sectional, n:311 65 + aged n: 169	China	BMI WHtR* CI	In older individuals, the highest association for OSAS developing in relation to adiposity was associated with waist/height ratio (R^2^ = 0.296)
	Wang et al. 2023 [71]	Cross-sectional, n: 5277 ≥ 60 aged	China	BMI WHtR BRI ABSI WWI* CI	All central obesity indices such as WWI, ABSI and CI were found to be associated with dementia and Alzheimer’s disease But **WWI stands out by showing a linear association with the likelihood of dementia and Alzheimer’s**
	Zhang et al. 2023 [72]	Cross-sectional, n:36,784 grouped for those over 60 years of age	China	BMI WHtR BRI*	**BRI has higher sensitivity and specificity for predicting low glomerular velocity** (sensitivity 54.3%, specificity 65.4%) In the group aged 60 years and older, **higher BRI** (Q3 and Q4) **was associated with lower glomerular filtration rate**
	Lin et al. 2023 [73]	Cross-sectional, n: 8154 (> 60 aged) grouped for those over 60 years of age	USA	BRI* ABSI	For ABSI, **there is no significance in any model for the risk of kidney stone development** in the > 60 years age group for both sexes, **High BRI increases the risk of developing kidney stones** by 23% in women (OR: 1.23 1.04–1.46), but not in men, adjusting only for race
	Feng et al. 2019 [74]	Cross-sectional, n:570 Mean age: 62,3 ± 6,5 year	China	BAI BRI* AVI WHtR *	**BRI can be used as an alternative anthropometric index to detect insulin resistance** (Men: sensitivity 82%, specificity 63% Women: sensitivity 72%, specificity 72%) However, the best predictors of insulin resistance are AVI in men (OR 19.73, 95% CI 2.51–155.04, P < 0.01) and BMI in women (OR 15.55)
	Yang et al. 2023 [75]	Cross-sectional, n:2,601 ≥ 45 aged	China	WHtR* BRI* ABSI CI	**Low BRI and WHtR associated with normoglycemia**
	Bai et al. 2022 [76]	Cross-sectional, n: 69,388 ≥ 60 aged Mean age: 71,4 ± 6,9	China	WHtR BRI BMI*	**High WHtR, BMI and BRI** are **positively associated with T2DM in both men and women** (Waist/height ratio and BRI are highly correlated with BMI r = 0.996)
	Kim et al. 2022 [23]	Cross-sectional, n: 2031 Mean age: 75.9 ± 3,9 yıl	Korea	BMI WHtR WWI*	**The WWI is the only index that is negatively correlated with physical function in both men and women**, can diagnose sarcopenic obesity in men, while this effect is not reported in women
	Park et al. 2023 [53]	Cohort, n:515	Korea	WWI* WHtR	Both indices show a high correlation with visceral adiposity, but **WWI is also negatively correlated with appendicular muscle mass** (r = − 0.272), making it an effective anthropometric index for screening sarcopenic obesity
	Kim et al. 2021 [77]	Cross-sectional, n: 602 65 + aged Mean age: 72.1 ± 4,8 age	Korea	BMI, WWI*	**WWI correlated positively with total abdominal fat area** (r = 0.421), visceral fat area (r = 0.264) and total fat percentage (r = 0.465), and negatively with appendicular skeletal muscle mass (ASM) (r = − 0.511) and ASM/height2 (r = − 0.324)
Undernutrition	Başıbüyük 2021[6]	Cross-sectional, n: 2721 65 + aged: 55,9%	Turkey	BMI WHtR BRI BAI AVI*	Each can be used to assess the risk of malnutrition (AUC > 0.7). However, **the best predictors in both sexes are AVI, WHtR, BRI and BAI, respectively**
	Thuayngam et al. 2023 [78]	Cross-sectional, n: 347 60 + aged Mean age: 66,4 ± 5,3 yıl	Thailand	BMI Demiquet* Mindex*	**Mindex and Demiquet values are positively correlated with MNA scores and BMI**
	Malazonia et al. 2019 [79]	Cross-sectional, n: 75 60–74 aged	Georgia	BMI Demiquet* Mindex*	**Demiquet and Mindex can be used as BMI alternatives** (r = 0.979) (kappa = 0.8518)

*Refers to the anthropometric index with the highest correlation, those in bold type show the highlighted index and finding

*BMI *Body mass index, *WHtR *Waist to Height ratio, *BRI *Body roundness index, *BAI *Body adiposity index, *ABSI *A body shape index, *CI *Conicity İndex, *AVI *Abdominal Volume İndex, *WWI *Weight adjusted waist index, *MNA *Mini Nutritional Assesment, *AUC *Area Under the Curve

**Fig. 1:**
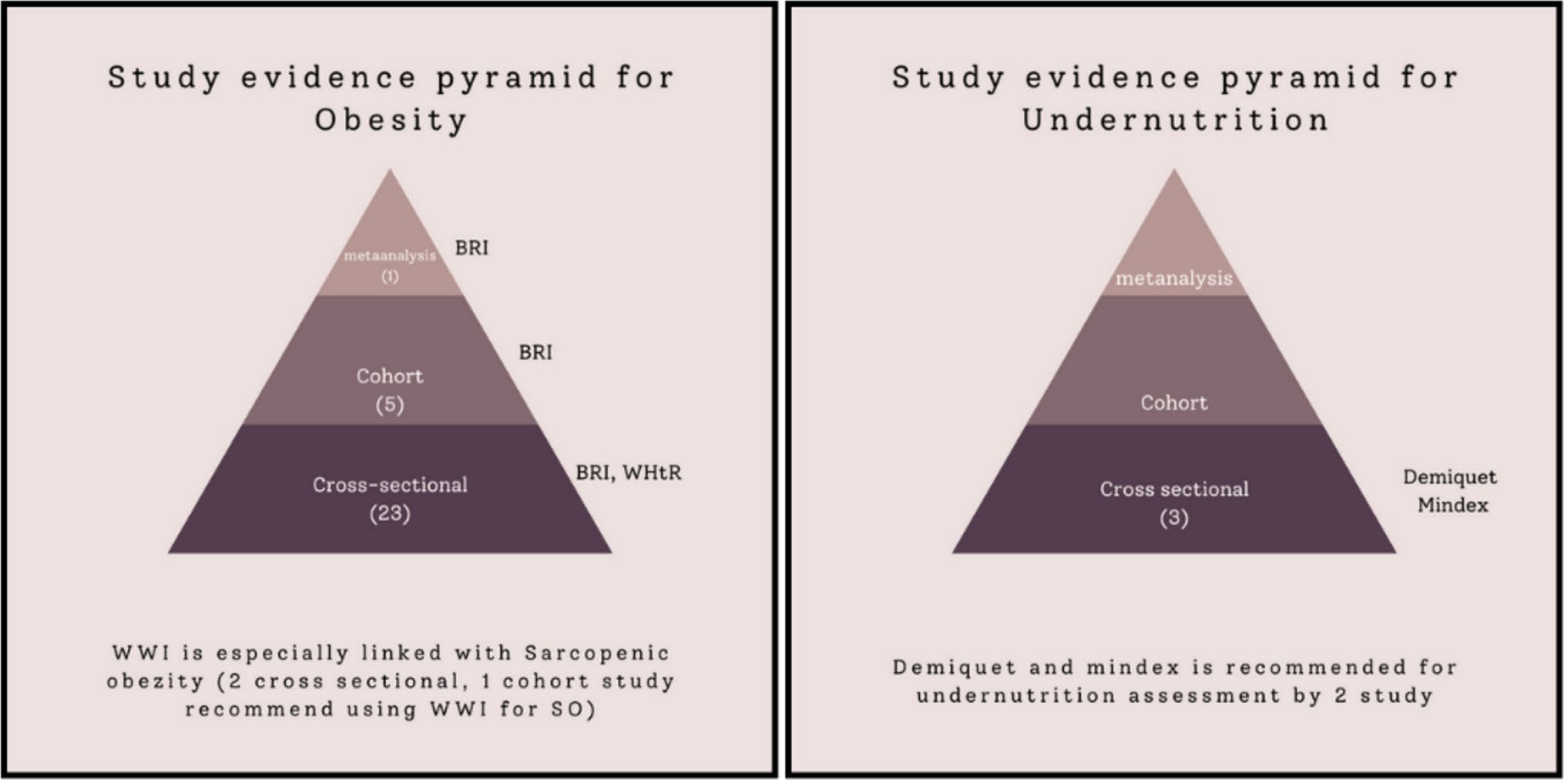
Classification of studies according to the evidence pyramid for malnutrition and prominent indices. BMI: Body mass index, WHtR: Waist to Height ratio, BRI: Body roundness index, BAI: Body adiposity index, ABSI: A body shape index, CI: Conicity İndex, AVI: Abdominal Volume İndex, WWI: Weight adjusted waist index

An analysis of studies conducted over the past five years indicates that not all anthropometric indices were evaluated in each study, and due to the limitations inherent in studying the older population, the assessments of predictive power remain incomplete. Although indices such as the BRI for predicting obesity and the Demiquet and Mindex for predicting malnutrition have emerged as prominent, determining ideal anthropometric indices is challenging due to changes in body composition, redistribution of subcutaneous fat, and the complex biology of adipose tissue in aging populations [9]. As previously mentioned, assessing height in older individuals can be inaccurate; therefore, estimating height through anthropometric measurements such as ulna length, knee height, or demi-span is encouraged, or alternatively, using indices that do not rely on height measurements (such as WWI, AVI, Demiquet, and Mindex) can be implemented [49, 54, 77]. Selecting the appropriate anthropometric index based on the specific variable being assessed is crucial. For example, the use of the WWI is particularly advantageous in the older people due to the high prevalence of sarcopenic obesity and the fact that it does not involve height measurements [77]. Additionally, the Demiquet and Mindex indices are advantageous due to their simplicity in screening and evaluation, and their alignment with malnutrition parameters [54]. The studies evaluated in terms of malnutrition classification, the number of studies according to the evidence pyramid, and the prominent anthropometric indices are illustrated in Fig. 1. A total of 32 studies comparing anthropometric indices within the scope of malnutrition were reviewed. Of these, 29 were related to obesity and 3 to undernutrition. When ranked according to the evidence pyramid, the BRI emerged as prominent in both meta-analyses (= 1) and cohort studies (= 5), while in cross-sectional studies, both the BRI (= 10) and the WHtR (= 9) were identified as effective indices. In the context of sarcopenic obesity, the use of the waist-to-weight ratio was recommended in all reviewed studies (= 3). For undernutrition, the majority of the reviewed studies reported that the use of the Demiquet and Mindex indices is advantageous in the older adults (*n* = 2).

## Conclusıon

This study is important in comparing anthropometric indices that can be used as predictors of malnutrition in the older population. However, the included studies were conducted on different populations and races. It should be noted that anthropometric indicators are influenced by factors such as race, age, and gender, and that body composition varies according to population and ethnic origin. Gender-related hormonal factors and aging also influence anthropometric indices and their cutoff points [80]. Population-based studies conducted to determine cutoff points have yielded different results. It is emphasized that these differences in results may stem from both the ethnic composition and the age average of the study population [58, 62].

Moreover, variations in measurement techniques and study designs may contribute to discrepancies in cutoff values. Differences in body composition assessment methods, such as BIA, DXA, and skinfold thickness measurements, could lead to inconsistent results across studies. Additionally, small sample sizes may limit statistical power and reduce the generalizability of findings. In light of this data, larger, population-based studies with standardized methodologies are needed to establish more reliable and applicable cutoff points.

Several potential confounding factors, such as age-related changes in body composition, sex differences in fat distribution, and ethnic variability in anthropometric characteristics, may influence the predictive power of these indices. Socioeconomic status, dietary patterns, and physical activity levels could also contribute to variations in cutoff values across populations. Addressing these factors is essential for a more accurate interpretation and application of population-specific anthropometric indices.

## Key References

- Liu C, Wong PY, Chung YL, et al. Deciphering the “obesity paradox” in the elderly: A systematic review and meta analysis of sarcopenic obesity. Obesity Reviews. 2023;24(2):e13534.
  - ○ This study highlights that the source of the obesity paradox in the *older people* is the combination of a high-fat percentage and muscle mass. It is emphasized that in obese individuals with low muscle mass, mortality is still increased, underscoring the importance of muscle mass.
- Park MJ, Hwang SY, Kim NH, et al. A novel anthropometric parameter, weight-adjusted waist index represents sarcopenic obesity in newly diagnosed type 2 diabetes mellitus. Journal of Obesity & Metabolic Syndrome. 2023;32(2):130.
  - ○ This study highlights that the WWI demonstrated the highest area under the receiver operating characteristic curve for detecting sarcopenic obesity (SO), which is commonly observed in the *older people*, making it a preferable anthropometric index for predicting SO. Additionally, the WWI may serve as a crucial tool for identifying cardiometabolic risk factors, particularly in older individuals.
- Volkert D, Beck AM, Cederholm T, et al. ESPEN practical guideline: Clinical nutrition and hydration in geriatrics. Clinical Nutrition. 2022;41(4):958–989.
  - ○ The study emphasizes the importance of individualized, comprehensive interventions as part of a multimodal, multidisciplinary approach to nutrition. It provides evidence-based recommendations for addressing malnutrition in older adults, highlighting the need for routine malnutrition screening. By advocating for a collaborative care model, the study offers healthcare providers a clear path to effectively prevent and treat malnutrition. Ultimately, the study underscores the critical role of screening in both the prevention and treatment of malnutrition, making it especially valuable for improving the health and well-being of older adults in clinical practice.

## Data Availability

No datasets were generated or analysed during the current study.
