# Correction: Evaluating Anthropometric Indices for Malnutrition Assessment in Older Adults: Scoping Review

**DOI:** 10.1007/s13668-025-00661-0

**Published:** 2025-05-22

**Authors:** Serife Akpinar Senture, Eda Koksal

**Affiliations:** Faculty of Health Science, Department of Nutrition and Dietetic, Gazi University, Emek, Bişkek Main St. 6. St No: 2, Çankaya, Ankara 06490 Turkey

**Current Nutrition Reports (2025) 14:65**

10.1007/s13668-025-00654-z

In the original version of this article, the Abstract section and Keywords are missing. It should have read as follows:

Abstract

Purpose of Review

The demographic transition in both global and national populations indicates a growing trend in the older population. This burgeoning older population brings about constraints marked by the effective presence of malnutrition alongside functional and physiological changes. Conducting screenings and assessments to prevent malnutrition and enable early intervention is crucial. In epidemiological and clinical studies, these screenings and assessments are often conducted through anthropometric measurements and a range of indices derived from them, encompassing both traditional ones like BMI (Body Mass Index) and newer ones like BRI (Body Roundness Index), BAI (Body Adiposity Index), ABSI (A Body Shape Index), CI (Conicity Index), WWI (Waist adjusted Weight Index), AVI (Abdominal Volume Index), Demiquet, and Mindeks. There is a lack of scrutiny regarding studies comparing these indices based on the type of malnutrition in the older population, hence this review aims to address the gap in the literature.

Recent Findings

Analysis of existing research reveals that BRI and waist-to-height ratio serve as predictors of obesity-related body fat quantity/distribution and metabolic risks, while WWI stands out in sarcopenic obesity, and Demiquet and Mindeks are prominent indices for screening inadequate nutrition.

Summary

Choosing anthropometric indices that do not include height in the older populations can have certain advantages. However, the selection of these indices should depend on the specific variable being assessed or screened such as obesity, malnutrition, or sarcopenic obesity.

Keywords: malnutrition, older adults, anthropometric indices, undernutrition, obesity, BRI (Body Roundness Index), BAI (Body Adiposity Index), ABSI (A Body Shape Index), Conicity Index (CI), Waist-adjusted Weight Index (WWI), Abdominal Volume Index (AVI), Demiquet, Mindeks

The original article has been corrected.

